# Methemoglobinemia: A Case Report

**DOI:** 10.7759/cureus.47752

**Published:** 2023-10-26

**Authors:** Mona J Malik, Muhammad Nabeel Pasha, Louisa Liu, Madlena Nalbandyan

**Affiliations:** 1 Internal Medicine, University of California Riverside, Riverside, USA; 2 Pulmonary and Critical Care Medicine, One Brooklyn Health, New York, USA; 3 Critical Care, University of California Riverside, Riverside, USA

**Keywords:** nadph, analgesics, hypoxia, nitrates, hyperbaric oxygen therapy (hbot), oxygen, blood, rave parties, confusion, methemoglobulinemia

## Abstract

Methemoglobinemia is a potentially life-threatening condition in which there is diminution of the oxygen-carrying capacity of circulating hemoglobin. It can result from either congenital or acquired processes. Methemoglobin forms when hemoglobin is oxidized to contain iron in the ferric (Fe3+) rather than the normal ferrous (Fe2+) state. Methemoglobinemia is a clinical diagnosis and is suspected in the presence of hypoxemia refractory to supplemental oxygen and the presence of chocolate-colored blood. Symptoms are usually dependent on methemoglobin levels; at levels higher than 35%, systemic symptoms from tissue hypoxia may be fatal. A high index of suspicion is required in patients with refractory hypoxia or cyanosis when treated with oxygen. Treatment options involve the removal of the inciting agent and treatment with the antidote methylene blue. Here we present a case of methemoglobinemia in a young patient who attended a college rave party.

## Introduction

Although hemoglobin holds and releases oxygen, a small part of hemoglobin is oxidized slowly to create methemoglobin which can hold but not release oxygen [1]. Daily methemoglobin creation is about 3% [1]. Acquired methemoglobinemia is a rare but potentially life-threatening condition [1]. Acquired methemoglobinemia can be caused by oxidative agents including sulfones, local anesthetics, and enhanced contents of nitrates in vegetables from unsuitable manure oil and well water. Methemoglobin levels of 10% or higher cause peripheral cyanosis. Methemoglobin levels higher than 35% cause generalized symptoms from tissue hypoxia [2]. Levels of 70% methemoglobin are potentially fatal and may result in coma [2]. At high levels, the condition is treated with hyperbaric oxygen, ascorbic acid, methylene blue, and riboflavin in high doses [3]. Herein, we present a case of methemoglobinemia in a young patient who attended a rave.

## Case presentation

A 20-year-old female with no significant past medical history was found unresponsive following a college rave party and was brought to the emergency department (ED). On presentation, she had a temperature of 97.8°F, blood pressure of 119/60, heart rate of 90 beats per minute (bpm), respiratory rate of 18, and oxygen saturation of 92 on 8 L/min O2 via nasal cannula. On exam, she had blue-grey skin discoloration and appeared lethargic but responsive, with no focal neurological deficits noted on physical examination. Her Glasgow Coma Scale score was 12. Pulmonary exam was clear to auscultation although the patient was short of breath with conversation and any exertion. Her initial labs including CBC, BMP, CXR, and EKG were unremarkable. Her urine color was blue, and her urine drug screen was positive for amphetamine and cocaine. Lactic acid was 5.8 mmol/L (N: < 2 mmol/L). Blood alcohol levels were at 207 mg/dL (N: 0- 400 mg/dL). Narcan was given immediately, with mild improvement in the patient's mentation. Her oxygen saturation remained 85-88% even while on the nonrebreather mask. Arterial blood gas (ABG) was measured (Table 1) on the nasal cannula first, and on the nonrebreather face mask subsequently.

**Table 1:** Arterial Blood Gas

O2 Device Settings	Cannula	Nonrebreather
Allen’s Test	POS (positive)	POS (positive)
pH (Art)	7.4	7.35
pCO2	31 mm Hg	37 mm Hg
pO2 (Art)	167 mm Hg	390 mm Hg
HCO3 (Art)	19.2 mEq/L	20.4 mEq/L
BE (Art)	-4.6 mEq/L	-4.6 mEq/L
FiO2 (Art)	32%	100%
tHb (Art)	13.0 gm/dL	15.2 gm/dL
O2 Hgb (Art)	95.10 %	29.10 %
COHb (Art)	<1.0 %	< 1.0%
MetHb (Art)	3.5%	30%
Corrected pCO2 (Art)	30 mm Hg	37 mm Hg
Corrected pH (Art)	7.41	7.35
Corrected pO2 (Art)	165 mm Hg	390 mm Hg
PaO2/FiO2 Calculated	522	390
Respiratory Rate	18	17

**Figure 1:**
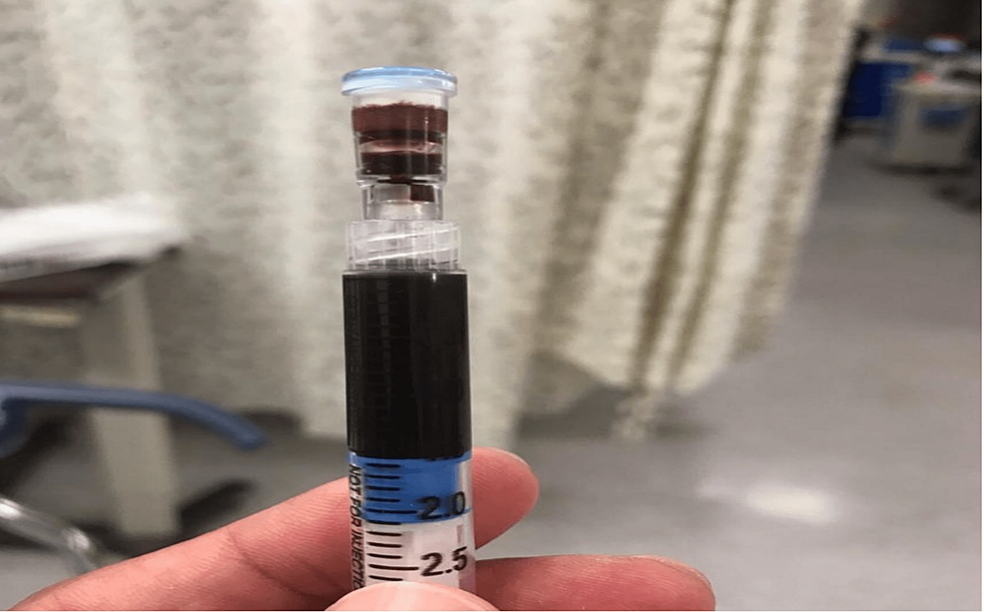
Arterial blood gas, on nonrebreather.

The blood was noted to be black in color (Figure 1), with a pH of 7.35, PCO2 of 37, bicarbonate level of 20.4 mmol/L, PO2 of 390, and a MetHb of 30%.

**Figure 2:**
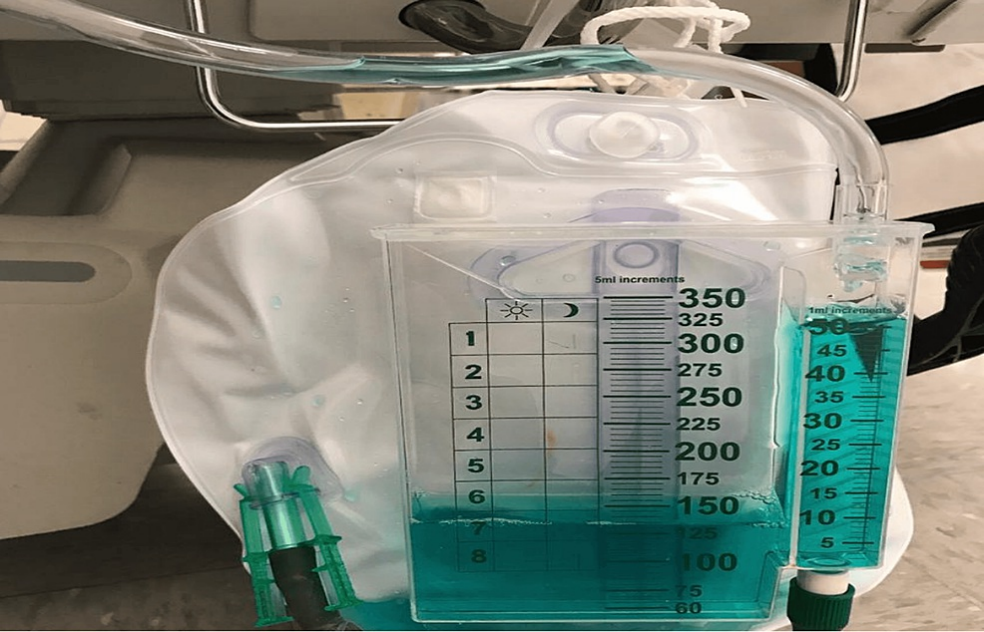
The patient's Foley bag with the classic blue hue of urine after methylene blue administration.

The patient was started on an infusion of methylene blue at 1 mg/kg over 15 minutes (Figure 2), and her oxygen saturation immediately improved.

Her mentation improved to baseline within eight hours. She informed us that she had taken Adderall and cocaine in addition to drinking alcohol. Repeat measurement of ABG showed a decline in PaO2 to 167 and a 3.5% MetHb level. Lactic acid levels had downtrended. The patient was fully alert with no neurological deficits thereafter. She did not require hospital admission and left for home from the ER.

## Discussion

Methemoglobinemia is a potentially life-threatening condition. It can occur with routine use of anesthetics, such as benzocaine and lidocaine. To our knowledge, there are 71 case reports on benzocaine-induced methemoglobinemia. The earliest reference was made in 1947 by Ocklitz reporting two infants treated with vaporized benzocaine powder for stomatitis. Infants have a higher level of fetal hemoglobin which is more easily oxidized to methemoglobin [3]. Infants also have lower NADH-methemoglobin reductase and glutathione peroxidase activity than adults. Infants have higher gastric pH, which favors the growth of bacteria such as E. coli and Pseudomonas aeruginosa that reduce nitrate to nitrite causing methemoglobinemia [3]. 

The acquired condition is caused by several offenders including household products, such as laundry detergent, bathroom and kitchen cleaning agents, and nail polish removers [1]. It may also be caused by the analgesic acetaminophen, which is one of the most commonly prescribed over-the-counter analgesics [1]. Other causes of methemoglobinemia include club drugs; among the most common of these are methylenedioxymethamphetamine (MDMA), also called ecstasy, and gamma-hydroxybutyrate (GHB), also known as liquid ecstasy. Furthermore, nitrite-producing bacteria, most commonly E. coli, Klebisiella, and Proteus also cause methemoglobinemia. In addition, nitrite-containing well water and manure oil [1, 4] can cause methemoglobinemia. 

Raves are a growing cause of methemoglobinemia beginning in the early '70s. Raves attendees have a 15% higher consumption of club drugs, compared to the general population [1]. Among the above-mentioned club drugs, cocaine is a growing cause of methemoglobinemia. Cocaine is seldom available in pure form, and usually has additives, most commonly local anesthetics or phenacetin. However, with cocaine, methemoglobinemia is related to adulterants such as local anesthetics, via oxidizing metabolites, or phenacetin, rather than to the cocaine itself. The association between phenacetin and the development of methemoglobinemia appears to be related to n-hydroxyphenacetin and p-phenetidine metabolites [1, 5]. 

Recently, nitrates have become popular among young adults. Nitrates are oxidizing agents, and when converted to nitrites, they oxidize hemoglobin, which converts into methemoglobin [2]. Adults with G6PD deficiency are at increased risk of developing methemoglobinemia as they have lower levels of nicotinamide adenine dinucleotide phosphate hydrogen (NADPH) and a higher chance of oxidation of nitrates to nitrites [3].

At methemoglobin levels below 15%, there are typically no symptoms [1]. The signs and symptoms of methemoglobinemia become prominent at MetHb levels of 15-20% or higher [1]. Pulse oximetry is consistent with lower oxygen saturation levels and the appearance of blood may be dark brown [1]. At levels between 15-20%, there may be cyanosis, with no improvement with supplemental oxygen [2]. Our patient's MetHb level on the arterial blood gas was 30% on nonrebreather oxygen supplementation. She exhibited central nervous system symptoms, with the presence of generalized weakness, dizziness, and altered mental status. At MetHb levels between 20-50%, there may be dyspnea, and central nervous system involvement with headaches, dizziness, syncope, and generalized fatigue and weakness, as was seen with our patient [3]. At even higher levels of 50-70%, there may be tachypnea, metabolic acidosis, dysrhythmia, seizure, and coma [3]. At levels > 70%, there may be death. [3]

Pulse oximetry is inaccurate in patients with methemoglobinemia, as the pulse oximeter reading is based on the assumption of only two varieties of hemoglobin present, which are oxyhemoglobin and deoxyhemoglobin [4]. CO-oximetry gives accurate concentrations because it can identify the absorptive characteristics of several hemoglobin species at different wavelengths, including methemoglobin [4]. 

Mild cases of methemoglobinemia usually do not require any treatment. Supplemental oxygen treatment and removal of causative agent from exposure are sufficient [4]. Methylthioninium chloride (methylene blue) is the treatment of choice for patients with symptomatic methemoglobinemia [5]. It acts as a substrate for NADPH-MetHb reductase, resulting in the formation of reduced methylthioninium chloride, reducing Iron (Fe) to its ferrous state [5]. The recommended dosage is 1-2 mg/kg [5].

## Conclusions

A high index of suspicion for methemoglobinemia should be maintained in cases of refractory hypoxia or cyanosis despite treatment with supplemental oxygen, especially in the setting of exposure to a known oxidative agent. Diagnostic clues are the presence of chocolate-colored blood and an oxygen saturation gap. Methylene blue remains the first-line treatment. 
